# Embodying self-compassion within virtual reality and its effects on patients with depression

**DOI:** 10.1192/bjpo.bp.115.002147

**Published:** 2016-02-15

**Authors:** Caroline J. Falconer, Aitor Rovira, John A. King, Paul Gilbert, Angus Antley, Pasco Fearon, Neil Ralph, Mel Slater, Chris R. Brewin

**Affiliations:** **Caroline J. Falconer**, PhD, Clinical Educational & Health Psychology; **Aitor Rovira**, MSc, Department of Computer Science; **John A. King**, PhD, Clinical Educational & Health Psychology, University College London, London, UK; **Paul Gilbert**, PhD, Mental Health Research Unit, University of Derby, Derby, UK; **Angus Antley**, PhD, Department of Computer Science; **Pasco Fearon**, PhD, Clinical Educational & Health Psychology, University College London, London, UK; **Neil Ralph**, DClinPsy, Clinical Educational & Health Psychology, University College London, London, and iCope Camden and Islington Psychological Therapies Service, London, UK; **Mel Slater**, DSc, Institució Catalana de Recerca i Estudis Avançats, University of Barcelona, Barcelona, Spain, and Department of Computer Science, University College London, London, UK; **Chris R. Brewin**, PhD, Clinical Educational & Health Psychology, University College London, London, UK

## Abstract

**Background:**

Self-criticism is a ubiquitous feature of psychopathology and can be combatted by increasing levels of self-compassion. However, some patients are resistant to self-compassion.

**Aims:**

To investigate whether the effects of self-identification with virtual bodies within immersive virtual reality could be exploited to increase self-compassion in patients with depression.

**Method:**

We developed an 8-minute scenario in which 15 patients practised delivering compassion in one virtual body and then experienced receiving it from themselves in another virtual body.

**Results:**

In an open trial, three repetitions of this scenario led to significant reductions in depression severity and self-criticism, as well as to a significant increase in self-compassion, from baseline to 4-week follow-up. Four patients showed clinically significant improvement.

**Conclusions:**

The results indicate that interventions using immersive virtual reality may have considerable clinical potential and that further development of these methods preparatory to a controlled trial is now warranted.

**Declaration of interest:**

None.

**Copyright and usage:**

© The Royal College of Psychiatrists 2016. This is an open access article distributed under the terms of the Creative Commons Attribution (CC BY) licence.

Self-criticism is known to be one of the major psychological factors that creates vulnerability and influences recovery and maintenance of depression.^1,2^ Innovative and easily disseminable interventions that address this, and in particular support long-term change for individuals, are urgently needed. Immersive virtual reality can potentially provide this and we report a proof-of-concept study applying one aspect of this technology, virtual embodiment.

When a life-sized virtual body substitutes a person's real body in immersive virtual reality, it typically generates an illusion of body ownership over the virtual body. The illusion of body ownership is the perceptual illusion that the body is one's own and relies on a first-person perspective view of the body^3,4^ and synchrony between touches seen on the virtual body and felt on the real body^5^ or synchrony between real and virtual body movements through real-time motion capture.^6^ Recent evidence shows that embodiment has a variety of physiological and psychological consequences that indicate that the person has identified with or taken on attributes of the virtual body, including changes in size perception after embodiment in a child body, and changes in implicit racial attitudes after embodiment in a body with a different skin colour.^7–10^

We previously developed a scenario designed to exploit the potential of virtual embodiment to increase feelings of self-compassion in healthy individuals high in self-criticism.^11^ In the first phase of our immersive virtual reality scenario, participants interacted compassionately with a crying virtual child while embodied in a virtual adult body. In the second phase, one group of participants then embodied the child body and could experience a recording of their compassionate gestures and words being delivered to them from this (child) embodied first-person perspective. By having participants embody an adult and then a child virtual body in succession, our scenario effectively provided a self-to-self situation enabling participants to deliver compassionate sentiments and statements to themselves. Consistent with predictions, this condition resulted in a significantly greater increase in self-compassion than a control condition in which participants saw the same gestures and heard the same words but from a non-embodied, third-person perspective. Self-criticism decreased significantly in both conditions.^11^ The current study uses an uncontrolled case series to assess whether this methodology could also potentially be of benefit in clinical depression.

## Method

### Experimental design and participants

The study utilised a case series with varying baseline length prior to the intervention. Patients were recruited from a local National Health Service Improving Access to Psychological Therapies service. Inclusion criteria were: meeting DSM-IV criteria for current major depressive disorder, according to the Structured Clinical Interview for DSM-IV,^12^ and reporting depressive symptoms at a stable level prior to treatment. Exclusion criteria were: psychotic disorders, organic brain disease, high risk of self-harm or suicide, current substance misuse and lack of fluency in English. Of 18 eligible patients who started treatment, 1 did not complete due to time commitments and 1 did not complete because she found hearing her voice played back to her aversive.

The sample comprised ten women and five men, with an average age of 32 years (range 23–61). All were White except for one Asian male. Four patients had had 1–2 previous episodes of depression, and the remainder reported more episodes with an average of 4 (overall range 1–6). Eight patients reported the onset of their first episodes in their late teens, five in their early 20s, and two in their 30s. Ten patients were currently on antidepressant medication, seven were currently receiving psychological therapy, seven were awaiting therapy and one had finished a course of therapy. One patient had comorbid attention-deficit hyperactivity disorder, one comorbid obsessive–compulsive disorder, and one comorbid obsessive–compulsive disorder and an eating disorder.

### Measures

#### Severity of depression

The primary outcome measure was the Patient Health Questionnaire-9 (PHQ-9),^13^ a measure often used to assess outcomes in psychological treatments for depression.^14^ It assesses symptoms over the previous 2 weeks and comprises 9 items scored 0 (‘not at all’), 1 (‘several days’), 2 (‘more than half the days’) or 3 (‘nearly every day’). A reduction of at least 5 points indicated reliable change and this reduction plus a follow-up score of 9 or less indicated clinically significant change.^15^ More transient fluctuations in depressed mood during the baseline and intervention periods were assessed with the Zung Self-Rating Depression Scale (SDS).^16^ The Zung SDS comprises 20 items scored 1 (‘a little of the time’), 2 (‘some of the time’), 3 (‘good part of the time’) or 4 (‘most of the time’) and was modified to be rated with respect to the previous 2 days.

#### Self-Compassion and Self-Criticism Scale

The Self-Compassion and Self-Criticism Scale (SCCS)^17^ consists of five scenarios that are potentially self-threatening and can elicit varying degrees of self-criticism or self-compassion (e.g. ‘You arrive home to find that you have left your keys at work’). Participants are instructed to imagine that these scenarios are happening to them now and rate on 7-point scales from 1 (‘not at all’) to 7 (‘highly’) the extent to which they would react to themselves in a harsh, contemptuous, critical, soothing, reassuring, and compassionate manner. The positive ratings are summed to generate the Self-Compassion Scale (range 15–105) and the negative ratings generate the Self-Criticism Scale (range 15–105). The scale has good internal reliability with Cronbach's alphas of 0.91 and 0.87 respectively, and is sensitive to change.^11,17,18^ Mean scores on self-criticism at baseline were markedly elevated relative to the healthy development sample described by Falconer *et al*,^17^ *t*(426)=3.11, *P*=0.002. In contrast, self-compassion scores in the patient sample were comparable with the healthy sample, *t*(426)=0.52, *P*=0.60.

#### Fears of Compassion Scales

Three related scales assess traits consisting of fear of experiencing compassion for oneself (15 items: example, ‘I fear that if I am more self-compassionate I will become a weak person’), fear of receiving compassion from others (13 items: example, ‘When people are kind and compassionate towards me I feel anxious or embarrassed’) and fear of experiencing compassion for others (10 items: example, ‘People will take advantage of me if they see me as too compassionate’).^19^ All items are rated on a 5-point scale from 0 (‘don't agree at all’) to 4 (‘completely agree’). High internal reliability (coefficient alpha 0.78–0.85) and validity data have been reported. Comparing this sample's mean scores at baseline with those of the students reported by Gilbert *et al*^19^ indicated that patients had greater fear of compassion for self, *t*(15.1)=4.25, *P<*0.001, and fear of compassion from others, *t*(14.7)=4.62, *P<*0.001, but less fear of compassion for others, *t*(15.1)=−2.65, *P<*0.05.

#### Virtual Reality Experience Questionnaire

This questionnaire (Appendix) was designed to assess the experience of being in the virtual environment. The question used to assess presence was based on previous virtual reality experiments.^20^ The illusion of body ownership as an adult and as a child were based on two questions (adult session 1 intercorrelation *r*(15)=0.69, *P* =0.004; child session 1 intercorrelation *r*(15)=0.78, *P*=0.001), adapted from questions used in earlier virtual reality studies.^8,9^ Other questions corresponded to aspects of the compassion scenario: recognising the adult as oneself when embodied in the child (3 items, session 1 coefficient alpha = 0.66), and the experience of feeling comforted when embodied in the child (3 items, session 1 coefficient alpha = 0.68). All items were scored on a scale ranging from −3 (‘not at all’) to +3 (‘very much so’), except for the single item on presence where −3 represented ‘(I felt like I was in) the lab taking part in an experiment’ and 3 represented ‘(I felt like I was in) a room where there was a child crying’.

### Virtual environment

The virtual room was created using Autodesk 3ds Max. It was an accurate 3D model of the actual room where the experiment was carried out. The virtual furniture (curtain and door) also matched the placement and size of the actual furniture. The only addition was a virtual mirror where the participants could see a reflection of their virtual body in the virtual environment. The virtual characters used, both adult and child, were acquired from Rocket box Studios. The scenario was implemented with the Unity 3D 4 game engine.

The participant's head was tracked with a 6-DOF InterSense IS-900, a high-rate low-latency tracking system that allows the system to know the participant's head position and orientation and thus adjust the imagery to their perspective in real time. The rest of the body was tracked with Natural Point's Optitrack system with 37 light reflective passive markers attached to the participant's body. Twelve V100 infrared Optitrack cameras captured the tracking volume and body suits from Natural Point were used. To deliver the 3D imagery, we used an nVidiaQuadro 4000 graphics card and annVis Sx111 head-mounted display (HMD). It has two screens built in with a resolution of 1280 × 1024 each. They provide a total horizontal field of view of 102 degrees. The HMD is designed in such a way that each eye can only see one of the screens. On each frame of the video, the scene is rendered twice, once on each screen taking into account the disparity between the eyes, providing a stable, immersive percept with stereoscopic depth.

### Procedure and materials

Participants were screened to determine whether they met study inclusion and exclusion criteria. They then attended an initial session to acquaint them with the virtual reality equipment. They were also presented with a video of the researcher participating in the virtual reality task (Supplementary Video S1).^11^ During this session, they completed the Zung SDS, PHQ-9, SCCS and Fears of Compassion Scales. They were then randomly assigned to baseline measurement periods of 4, 5, 6, 8 or 10 days, on each of which they completed the Zung SDS. Prior to commencing the study, the average Zung SDS score was 51.47 (s.d.=7.90) and the average PHQ-9 score was 17.33 (s.d.=3.42), consistent with the presence of major depression.^13^

In the second session, participants were given information concerning compassion and task instructions that presented a staged approach to reducing distress based on current knowledge and practice in compassion-focused therapy.^21^ As in Falconer *et al*^11^ they were provided with generic sentences that corresponded to each of these three stages (validation, redirection of attention and memory activation) and instructed to memorise them as best they could so as to deliver them slowly, softly and compassionately to the child. Participants practised their lines with the researcher to ensure that they were comfortable expressing themselves. After this, participants were fitted with the HMD and body tracking suit. Embodiment was achieved through a series of simple exercises lasting approximately 2 min in which they confirmed that the virtual body moved in synchrony with their own movements (visual–motor synchrony) while looking towards themselves directly or in the mirror. Patients were then introduced to the first phase of the scenario, which also lasted approximately 2 min. In this phase, the child was programmed to respond positively to the patient after the delivery of each stage of their compassionate response. The child movements and audio were previously recorded in conjunction with our three-stage compassionate response using a female actor. After their compassionate response, participants were asked to close their eyes to complete the body ownership questions, which were recited to them and their responses were recorded by the researcher.

In the second phase of the scenario, patients were embodied in the child virtual body and were guided through the same exercises as before to become accustomed to their environment from this new perspective and to the different body. Patients then re-experienced their compassionate response from their new perspective as the child. This included a real-time replay for approximately 2 min of their original adult body delivering compassion, which portrayed their own body movements and voice. Patients were instructed to merely stand, look and listen to their compassionate response. Participants then exited the virtual reality and completed the virtual reality experience questions including the body ownership questions relating to their time as the child, and the SCCS.

The immersive virtual reality session was repeated a further two times at approximately weekly intervals. After the third session, the primary and secondary outcome measures were assessed. Four weeks after the last session, patients completed the PHQ-9 and SCCS. They were also asked to rate on a 7-point Likert scale from 1 (‘not at all’) to 7 (‘highly’) the extent to which they would recommend the study to others, and to comment on their experience of immersive virtual reality.

### Ethics statement

All procedures and materials were approved through the National Research Ethics Service (London–Stanmore section). Written informed consent was obtained from each patient prior to commencement of the study. All patients were given an inconvenience allowance of £36 for taking part in the study.

### Analysis

All variables were approximately normally distributed except for Zung SDS scores at the end of session 3. Changes in the experience of immersive virtual reality (e.g. degree of body ownership) over the three sessions were tested with a series of repeated-measures ANOVAs. To examine trends over time, the main effect test from the ANOVA was replaced by tests of linear and quadratic effects. Similar analyses were employed with the Zung SDS between start and end of baseline, after each intervention session, and at follow-up. For consistency, changes in all primary (PHQ-9) and secondary (SCCS, Fear of Compassion Scales) outcome variables were assessed between baseline, the end of the intervention and follow-up using repeated-measures ANOVA. We were unable to obtain end-of-intervention data from two participants. We therefore repeated the analyses on the entire sample, testing for changes between baseline and follow-up only. These yielded identical findings and are not reported further.

## Results

### Experience of immersive virtual reality

Already during session 1, answers to the presence item (median = 1; interquartile range (IQR) = 0 to +2) were comparable with previous studies.^8^ Answers at session 1 to the more specific questions on body ownership with respect to the adult body (median = 1.5, IQR = −0.5 to +2.0) and body ownership of the child body (median = 1, IQR = −1.5 to+1.5) were comparable with the single session results with self-critical students^11^ exposed to the same scenario. However, at the first session, scores on adult body ownership were significantly higher than corresponding scores on child body ownership, *t*(14) = 2.17, *P <*0.05. This was not the case at subsequent sessions, largest *t*(12) = 0.76, not significant.

Table 1 shows changes in the questions relating to presence, body ownership and experience of immersive virtual reality over the three sessions on the various −3 to +3 scales. The main change was a linear increase in recognition of the adult as oneself when embodied in the child, *F*(1, 12)=14.48, *P*=0.003, *η*_p_^2^=0.55. There were also linear increases in body ownership as the child, *F*(1, 12)=3.58, *P*=0.08, *η*_p_^2^=0.23, and in the experiences of being comforted while in the body of the child, *F*(1, 12)=3.60, *P*=0.08, *η*_p_^2^=0.23, but these were weaker and non-significant. There were no other linear or quadratic effects of interest, largest *F*(1, 14)=2.18, not significant.

**Table 1:** Means (standard deviations) of scales measuring the experience of three sessions of immersive virtual reality^a^

Experience of virtual reality scales	Virtual reality session		
	Session 1	Session 2	Session 3
Presence	0.77 (0.17)	0.23 (1.69)	0.69 (1.32)
Adult embodiment	0.88 (1.67)	1.00 (1.59)	1.19 (1.52)
Child embodiment	0.08 (1.87)	1.00 (1.37)	0.92 (1.82)
Recognise adult as self	−0.15 (1.14)	0.92 (1.22)	1.31 (0.70)
Feels comforted as child	0.82 (1.30)	0.82 (1.17)	1.28 (1.30)

a ‘Presence’ is the mean of 1 questionnaire item, ‘Adult embodiment’ and ‘Child embodiment’ are the mean of 2 questionnaire items, ‘Recognise adult as self’ is the mean of 3 questionnaire items, ‘Feels comforted as child’ is the mean of 3 questionnaire items.

Scores on adult and child body ownership were strongly positively correlated across all three sessions, smallest *r*(15)=0.73, *P*=0.002. Ratings of feeling comforted while in the body of the child were most consistently correlated with recognising the adult as oneself, although the correlation was not significant for session 3 (session 1: *r*(15)=0.58, *P*=0.02; session 2: *r*(14)=0.60, *P*=0.02; session 3: *r*(13)=0.35, *P*=0.25). On average patients said they would recommend the study strongly to others (mean=5.5, s.d.=1.35).

### Changes in severity of depression, self-criticism and self-compassion

Transient changes in depressed mood over the previous 2 days as measured with the Zung SDS were assessed prior to the study (*M*=54.80, s.d.=4.80) and again after the individual baseline period, after each immersive virtual reality intervention session, and at 4-week follow-up. A repeated-measures ANOVA found no evidence for linear, quadratic or cubic changes in transient mood over these six measurement points, largest *F*(1, 12)=2.27, *P*=0.16.

The primary outcome measure was the PHQ-9. Scores at baseline, after the immersive virtual reality sessions, and at 4-week follow-up are summarised in Table 2. There was a linear effect of time, *F*(1, 12)=14.04, *P=*0.003, *η*_p_^2^=0.54, such that participants became less severely depressed post-intervention but no significant quadratic effect, *F*(1, 12)=0.06, not significant. The within-participant effect size from baseline to follow-up controlling for the dependence between means was 1.11 (Cohen's *dz*). Of the 15 patients, 5 showed reliable improvement and a further 4 showed clinically significant change.

**Table 2:** Means (standard deviations) of primary outcome variables at baseline, post-intervention and 4-week follow-up

Measurements	Study time points		
	Baseline	Post-virtual reality intervention^a^	4-week follow-up
Patient Health Questionnaire-9	17.54 (3.43)	14.23 (6.03)	11.69 (6.18)
SCCS: self-criticism	67.23 (23.82)	49.54 (20.44)	34.38 (16.85)
SCCS: self-compassion	35.54 (19.03)	38.92 (19.28)	52.00 (16.29)
Fear of compassion for self	29.15 (11.57)	26.38 (8.56)	21.23 (10.51)
Fear of compassion for others	15.23 (8.53)	15.77 (9.58)	13.77 (9.71)
Fear of compassion from others	27.69 (9.77)	25.54 (8.03)	21.23 (7.95)

SCCS, Self-Compassion and Self-Criticism Scale.

a Data missing from two patients at this time point.

## Appendix

### Items measuring the experience of immersive virtual reality

#### Presence

I felt like I was in a room where a child was crying as opposed to in a lab taking part in an experiment.

#### Adult body ownership and agency

I felt as if the body I saw when I looked down was my own body.

I felt as if the body I saw when I looked in the mirror was my own body.

#### Child body ownership and agency

I felt as if the body I saw when I looked down was my own body.

I felt as if the body I saw when I looked in the mirror was my own body.

#### Child recognises adult as self

I felt like I was in the child's role.

I could recognise myself as the adult when I was the child.

I could recognise myself in the movement of the adult when I was the child.

#### Child sense of being comforted

I felt like I was giving myself compassion when I was the child.

I felt comforted by myself as the adult when I was the child.

I felt reassured by myself as the adult when I was the child.
